# Cancer stem cell fate determination: mito-nuclear communication

**DOI:** 10.1186/s12964-023-01160-x

**Published:** 2023-06-27

**Authors:** Mengchen Fan, Ying Shi, Jumei Zhao, Ling Li

**Affiliations:** 1 School of Basic Medical Sciences, Medical College of Yan’an University, Yanan, 716000 China; 2 Department of Cell Biology, National Translational Science Center for Molecular Medicine, State Key Laboratory of Cancer Biology, Fourth Military Medical University, Xi’an, 710032 China

**Keywords:** Cancer stem cells (CSCs), Mitochondria, Cell fate determination, Mito-nuclear communication

## Abstract

Cancer stem cells (CSCs) are considered to be responsible for tumor recurrence and metastasis. Therefore, clarification of the mechanisms involved in CSC stemness maintenance and cell fate determination would provide a new strategy for cancer therapy. Unregulated cellular energetics has been accepted as one of the hallmarks of cancer cells, but recent studies have revealed that mitochondrial metabolism can also actively determine CSC fate by affecting nuclear stemness gene expression. Herein, from the perspective of mito-nuclear communication, we review recent progress on the influence of mitochondria on CSC potential from four aspects: metabolism, dynamics, mitochondrial homeostasis, and reactive oxygen species (ROS).

Video Abstract

**Supplementary Information:**

The online version contains supplementary material available at 10.1186/s12964-023-01160-x.

## Introduction

Cancer stem cells (CSCs) are a subpopulation of cancer cells with the potential for self-renewal and multidifferentiation and thus drive carcinogenesis, chemoresistance, recurrence and metastasis [1–6]. CSCs are also known as cancer stem cell-like cells [7], tumorigenic cells [8], tumor stem-like cells (TSCs) [9], and cancer- or tumor-initiating cells (CICs or TICs) [10, 11]. In 1997, CSCs were successfully isolated from the blood of leukemia patients for the first time [12]. Subsequently, CSCs were further identified in solid tumors, such as colon cancer [13], breast cancer [14], skin squamous cell cancer [15] and glioblastoma (GBM) [16]. However, CSCs are as heterogeneous as cancer cells, include metastatic cancer stem cell (MeCSC) or chemoresistant cancer stem cell (CRCSC) subsets, and have a quiescent or proliferative status and epithelial or mesenchymal status [17]. In response to various stimuli, the subsets or status of CSCs can be altered accordingly; therefore, CSCs are also plastic [18]. Moreover, upon attack by chemo/radiotherapy [19–22], hypoxia [23] and detachment [24], cancer cells can acquire stemness potential. Thus, plasticity is an important feature of CSCs and the key point to understanding CSC stemness maintenance and fate determination.

Mitochondrial energy metabolism is essential to CSCs in various intracellular activities, especially for nuclear stemness gene expression. Under different functional statuses or environmental conditions, plastic CSCs might adopt different metabolic patterns accordingly. Thus, the acquisition of CSC potential is accompanied by a reprogramming of cellular metabolism [25, 26]. Furthermore, cellular metabolism has been reported to determine CSC fate by epigenetically modifying nuclear stemness genes *via* metabolites. Therefore, mitochondrial energy metabolism is not merely a phenotype of CSCs but also a determinant of CSC fate. In this review, we focus on the regulation of CSC plasticity from the view of mitochondrial signals, that is, the regulation of mitochondrial metabolism, dynamics, mitochondrial homeostasis, and reactive oxygen species (ROS) on CSC potentials and the involvement of mito-nuclear communication.

## Cancer stem cell plasticity and fate determination

The CSC theory holds that cancer cells are heterogeneous, and rare CSCs are the major driver of tumor initiation, metastasis and therapeutic resistance and thus the target for eradicating tumors [27–30]. More recently, the plastic CSC model has become widely accepted, and plasticity has become the main challenge of CSC targeted therapy [31]. Effective therapy must focus on the key regulatory factors that both maintain and induce the stemness of CSCs.

### Cancer stem cell plasticity

**Fig. 1:**
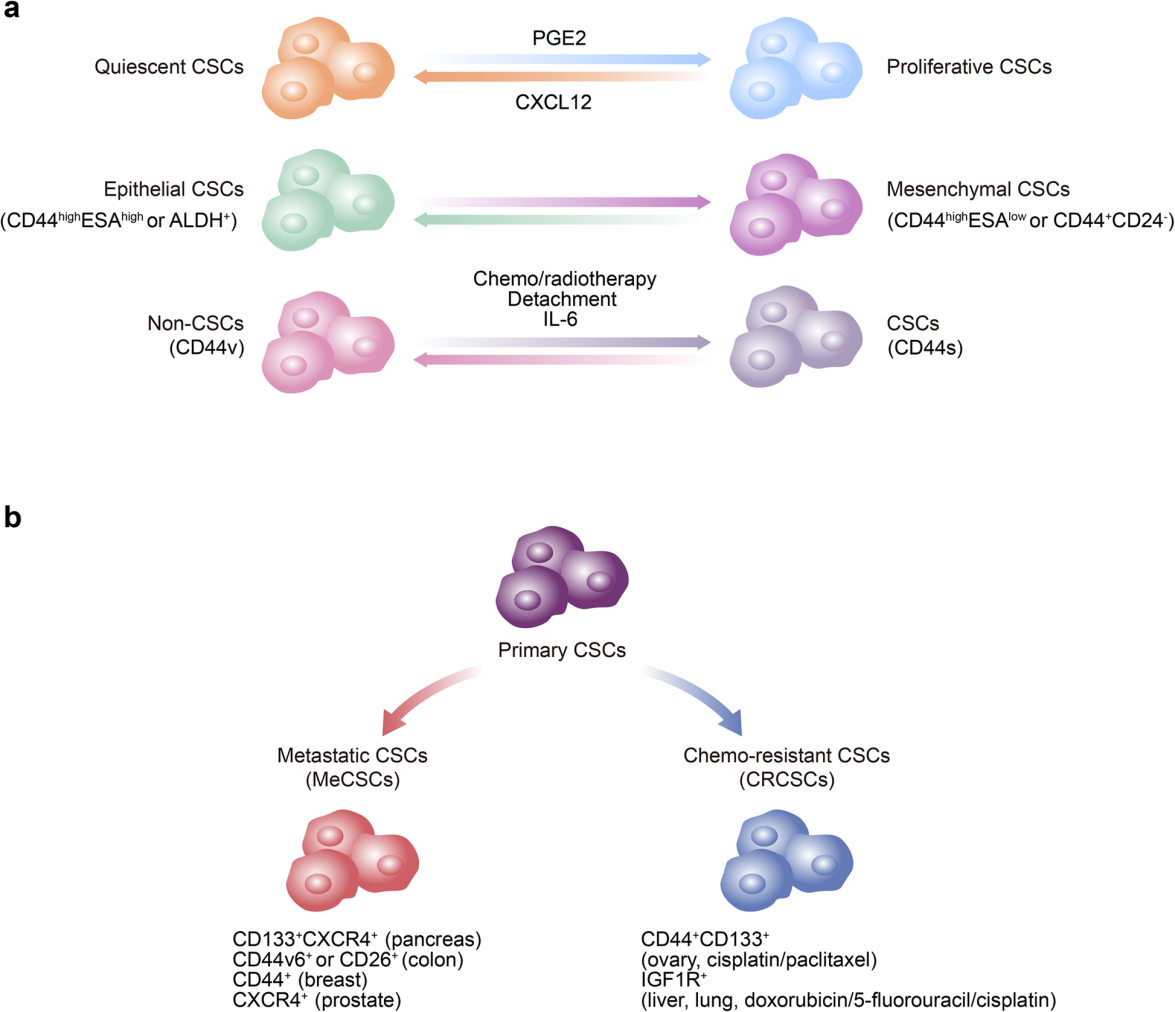
Phenotypic plasticity of cancer stem cells. The plasticity of CSCs is exhibited as the dynamic and reversible transitions between quiescent and proliferative CSCs, epithelial and mesenchymal CSCs and CSCs and non-CSCs (**A**) or the evolution from primary CSCs to MeCSCs or CRCSCs (**B**). *CSCs* Cancer stem cells, *MeCSCs* Metastatic cancer stem cells, *CRCSCs* Chemoresistant cancer stem cells

Cell plasticity is defined as the ability of cells to quickly adapt to the changing microenvironment by dynamically switching between different cellular statuses or phenotypes in a reversible manner, which hijacks the program of dedifferentiation or transdifferentiation in cells [32–34]. The plasticity of CSCs, however, is exhibited as the dynamic and reversible transitions between quiescent and proliferative CSCs, epithelial and mesenchymal CSCs, CSCs and non-CSCs, or the evolution from primary CSCs to MeCSCs or CRCSCs, which respond to the adverse tumor microenvironment (Fig. 1). Higher CSC plasticity is likely to facilitate tumor progression and is associated with poor patient clinical outcomes.

Due to changing microenvironment signals, CSCs are not always in a quiescent status but can be activated and enter a proliferative status and then initiate tumor growth and recurrence [35]. In bladder cancer, for example, combined treatment with gemcitabine and cisplatin (GC) induces the production of a high level of prostaglandin E2 (PGE2), which further forces CSCs to exit from a quiescent status and enter a proliferative status and then causes tumor progression [36]. In addition, Prx1+ mesenchymal progenitor-derived CXCL12 maintains leukemic stem cells (LSCs) in a quiescent and tyrosine kinase inhibitor (TKI)-resistant status, whereas CXCL12 deletion forces LSCs to enter a proliferative (cycling) status and makes them subsequently sensitive to TKI [37] (Fig. 1A).

Another important manifestation of CSC plasticity is the transition between epithelial-like phenotypes and mesenchymal-like phenotypes, that is, epithelial-to-mesenchymal transition (EMT). In squamous cell carcinoma, two different CSC subsets are found: CD44^high^ESA^high^ CSCs (non-EMT subset), which have an epithelial phenotype and proliferate rapidly; and CD44^high^ESA^low^ CSCs (EMT subset), which have a mesenchymal phenotype and migrate extensively [38]. When migrating to a secondary site, EMT CSCs recover into a proliferative mesenchymal-to-epithelial transition (MET) phenotype and then form metastatic tumors. Similarly, CD24^-^CD44^+^ breast cancer stem cells (BCSCs) located at the tumor invasive frontier display a quiescent mesenchymal phenotype, while aldehyde dehydrogenase-positive (ALDH^+^) BCSCs distributed more centrally show a proliferative epithelial phenotype. Moreover, BCSCs switch between a mesenchymal phenotype and an epithelial phenotype [39] (Fig. 1A).

Moreover, CSCs and non-CSCs can interconvert and achieve a dynamic balance [40, 41]. It has been reported that bulk breast cancer cells can convert into BCSCs under certain conditions, such as chemo/radiotherapy [42], detachment [24], and IL-6 induction [43]. During this process, breast cancer cells are switched between non-CSCs and CSCs by selective splicing of CD44 into CD44v or CD44s, and cancer cells with high levels of CD44v lose CSC stemness, while those with high levels of CD44s gain CSC stemness [44] (Fig. 1A).

In addition, CSCs exhibit phenotypic evolution during the process of tumor progression. During the early stage of tumorigenesis, primary CSCs are present in tumor cells with gene mutations. As a tumor develops into an advanced stage, MeCSCs with the potential to disseminate from the primary site, survive in the circulation, and seed and expand in the new microenvironment evolve (Fig. 1B). These MeCSCs can be organ specific [45]. When patients receive targeted therapy and/or chemotherapy, primary CSCs survive long-term administration of drugs; that is, CRCSCs or drug-resistant CSCs are developed [17] (Fig. 1B). It has been reported that MeCSCs share similar stemness potentials and epigenetic regulation mechanisms with primary CSCs. The first MeCSCs were identified as CD133^+^CXCR4^+^ subpopulations in CD133^+^ pancreatic CSCs with the potential to metastasize to the liver [46]. MeCSC subpopulations such as CD44v6 CSCs or CD26^+^ CSCs in colorectal cancer [47, 48], CD44^+^ CSCs in breast cancer [49], and CXCR4^+^ CSCs in prostate cancer [50] have also been found. Regarding CRCSCs, the CD44^+^CD133^+^ subpopulation with the potential for drug resistance was identified in CSC-like SKOV3 ovarian tumorspheres treated with cisplatin and/or paclitaxel [51]. In addition, IGF1R^+^ chemoresistant TSCs are found in lymphoma hepatocellular carcinoma (HCC) or Lewis lung cancer cells treated with doxorubicin, 5-fluorouracil or cisplatin [9] (Fig. 1B).

The plasticity of phenotype and status indicates that the developmental fate of cells can be changed; for example, it can be shifted toward a more undifferentiated status *via* dedifferentiation or to other lineages *via* transdifferentiation. Plasticity enables CSCs to better survive in adverse environments by easily switching their status and quickly altering phenotypes in response to various internal or external signals [52].

### Cancer stem cell fate determination

Although the alteration of plastic cellular phenotype or status is triggered by environmental cues, the decision of CSC fate is actually determined by the expression of nuclear stemness genes, which are regulated by specific transcription factors (TFs) [53]. In glioblastoma stem cells (GSCs), for example, the expression levels of key stemness-related TFs (POU3F2, SOX2, SALL2, and OLIG2) are significantly higher than those in more differentiated tumor cells. These four TFs can dedifferentiate and reprogram GBM cells into GSCs in *in vitro* cultured cells and *in vivo* animal models [54]. In contrast, dual-specificity tyrosine phosphorylation-regulated kinase 1A (DYRK1A) promotes the differentiation of GSCs and inhibits the acquisition of stemness potential by decreasing the expression of SOX2 [55].

With the gain or loss of stemness potential, the expression of stemness-related TFs is increased or decreased and is regulated at the posttranscriptional or translational level *via* epigenetic modifications. Growing evidence reveals that nonmutational and reversible epigenetic events, such as histone and chromatin modifications or DNA methylation, significantly contribute to CSC plasticity and carcinogenesis. For instance, SIRT1-mediated deacetylation of β-catenin maintains its stability, and the resulting nuclear accumulation of β-catenin increases the transcriptional level of NANOG and promotes the stemness potential of liver CSCs [56]. In another study, the zinc finger and homeobox 2 (ZHX2) protein was reported to eliminate liver CSC features by transcriptionally repressing KDM2A and inhibiting KDM2A-mediated demethylation of histone H3 lysine 36 in the promoter regions of stemness-related TFs (NANOG, SOX4, OCT4) [57].

Many factors inside and outside the cells, such as growth factors, inflammatory mediators, intracellular pH, mitochondrial metabolites and ROS, can influence the activity of stemness-related TFs at the level of epigenetic modification and then alter cell fates [58, 59]. Recently, an increasing number of studies have suggested that mitochondria play important roles not only in maintaining CSC stemness but also in determining CSC fate [60–66]. Therefore, we summarize CSC fate determination from a new view of mito-nuclear communication.

## Mito-nuclear communication and CSC fate determination

Mitochondria have their own genetic material (mtDNA) and corresponding gene transcription and protein translation systems. However, the majority (>99%) of proteins in mitochondria are not encoded by the mitochondrial genome but by the nuclear genome [67]. To harmonize nuclear-encoded protein synthesis with appropriate mitochondrial biogenesis or energy metabolism, the crosstalk between mitochondria and nucleus, in other words, mito-nuclear communication, has evolved. Proper communication between mitochondria and the nucleus allows mutual benefits and ensures the overall fitness of cells.

Ordinarily, mitochondria are under tight control by the nucleus through anterograde regulation signaling (from nucleus to mitochondria) according to cellular energy needs [68]. However, more recent studies have demonstrated that mitochondria can also generate retrograde signals to the nucleus *via* mito-to-nuclear communication mediated by small molecules, metabolites, peptides, mtDNA and ions through physical contact or signal transmission [69–71]. This concept expands the previous understanding that mitochondria are not merely semiautonomous organelles. Moreover, mitochondria can actively influence the expression of nuclear stemness genes, reprogram cell metabolism and phenotype, and thus determine CSC fate [68, 72, 73].

First, mitochondrial energy metabolism-derived metabolites or ROS are reported to play important roles in CSC fate determination through metabolic and epigenetic modification of stemness genes. In addition, mitochondrial dynamics or the balance between biogenesis and mitophagy could exert an influence on CSCs. In the following section, we discuss the influences of mitochondrial retrograde signaling on CSC fate determination in four aspects: energy metabolism, dynamics, mitochondrial homeostasis, and ROS.

### Mitochondrial energy metabolism

**Fig. 2:**
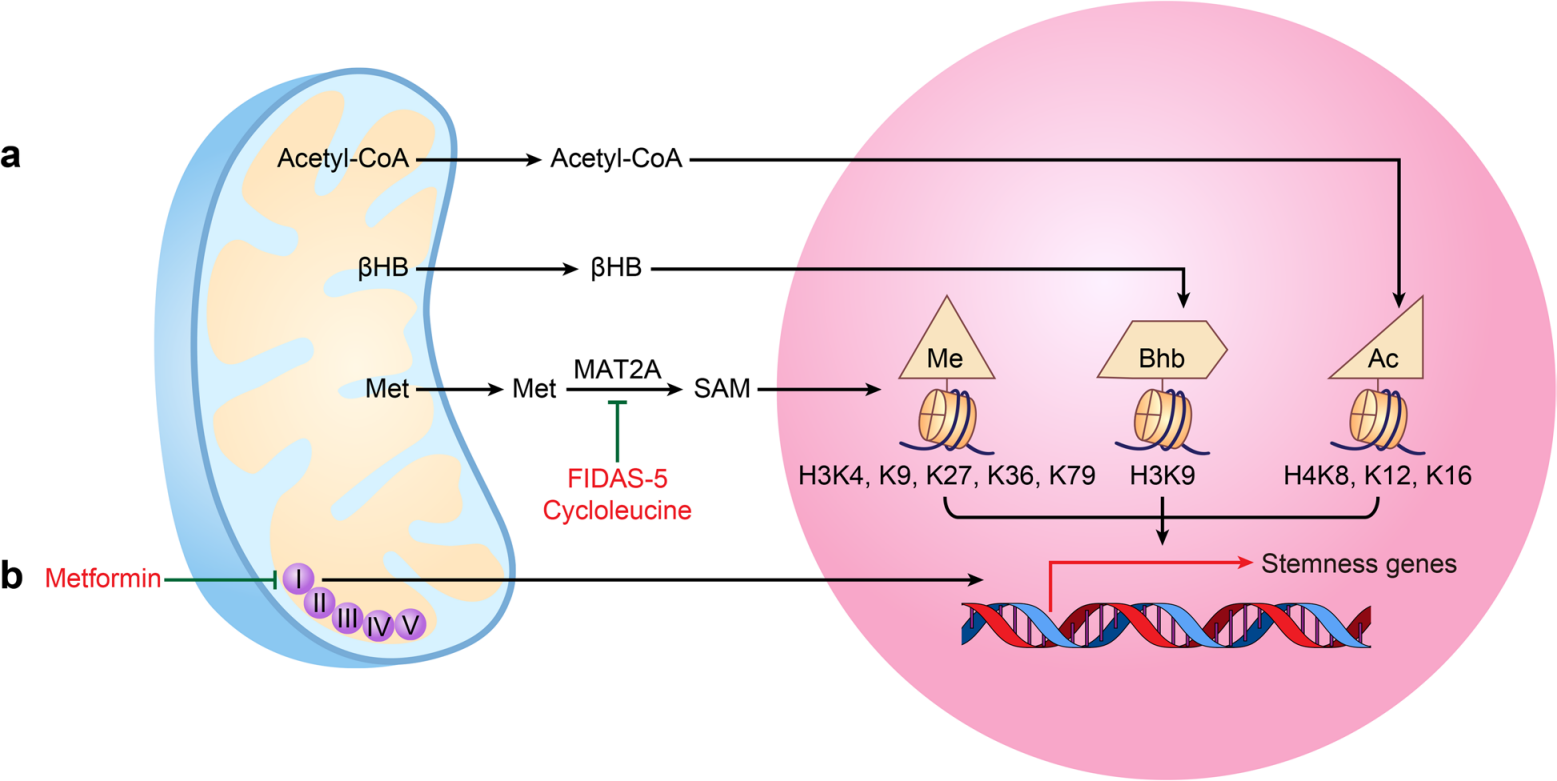
Mitochondrial energy metabolism and cancer stem cell stemness. **A** Acetyl-CoA promotes the expression of stemness-related transcription factors by enhancing the acetylation levels of histone H4K8ac, H4K12ac, and H4K16ac. βHB upregulates the expression levels of CSC marker genes by increasing the β-hydroxybutyrylation level of histone H3K9bhb. **B** Metformin suppresses mitochondrial complex I of oxidative phosphorylation and downregulates the expression of CSC-related genes. Inhibitors of the key methionine cycle enzymes MAT2A, cycloleucine and FIDAS-5 reduce CSC stemness by decreasing SAM levels and the expression of the methylation marks H3K4me3 alone or H3K9me3, H3K27me3, H3K36me2, H3K36me3 and H3K79me3. *acetyl-CoA* acetyl-coenzyme A, *βHB* β-hydroxybutyric acid, *CSC* Cancer stem cell, *TFs* Transcription factors, *MAT2A* Methionine adenosyltransferase 2A, *SAM* S-adenosylmethionine

Mitochondrial energy metabolism has profound impacts on the fate of CSCs [74–76] (Fig. 2). Recently, mitochondrial metabolites, which are generally involved in energy support, were reported to act as signaling molecules and to play critical roles in controlling gene expression [77]. In detail, mitochondrial metabolites, such as acetyl-coenzyme A (acetyl-CoA), β-hydroxybutyric acid (βHB), S-adenosylmethionine (SAM), NAD^+^, succinate, *α*-ketoglutarate (*α-*KG), ATP, and FAD, act as cofactors of epigenetic modifying enzymes and drive the acquisition of stemness potential by affecting stemness gene expression [72, 78]. For example, by enhancing the acetylation levels of histone H4K8ac, H4K12ac, and H4K16ac, acetyl-CoA activates the protein expression of stemness-related TFs (c-MYC, OCT4, KLF4, SOX2), which further increases the tumorsphere formation of CSCs in triple-negative breast cancer [79] (Fig. 2A). Furthermore, in hepatocellular carcinoma, accumulated βHB upregulates the expression levels of CD44, CD133, SOX9 and EpCAM by increasing the β-hydroxybutyrylation level of histone H3K9bhb and then significantly improves the proportions of CSCs for *in vivo* tumor formation and increases the abilities of colony formation and tumorsphere formation [80] (Fig. 2A). However, in acute myeloid leukemia (AML), low SAM levels decrease global DNA methylation, causing increased expression of differentiated myeloid genes (*CD11b*, *CD14*) but decreased expression of stemness genes, thereby enhancing differentiation but inhibiting self-renewal in LSCs [81].

### Mitochondrial dynamics

Mitochondrial dynamics, which involve vigorous changes in mitochondrial morphology between fission and fusion, are essential events that maintain the distribution, function and vitality of mitochondria [82–85]. Mitochondrial fission is the process by which a single mitochondrion divides into two short, round, balloon-shaped or fragmented daughter mitochondria and is mainly regulated by dynamin-related protein 1 (DRP1), DRP1 receptor mitochondrial fission factor (MFF), and fission factor 1 (FIS1) [86, 87]. Mitochondrial fusion is a phenomenon in which two closely contacted mitochondria are coordinately fused in the outer and inner membranes, forming an elongated, large, and interconnected mitochondrial network. The process of mitochondrial fusion is mainly regulated by the outer membrane fusion proteins mitofusins 1 and 2 (MFN1, MFN2) and the inner membrane fusion protein optic atrophy 1 (OPA1) [88]. To meet the cellular energy requirements in response to environmental changes, mitochondria dynamically switch between tubular and fragmented forms by balancing the process of fission and fusion.

**Fig. 3:**
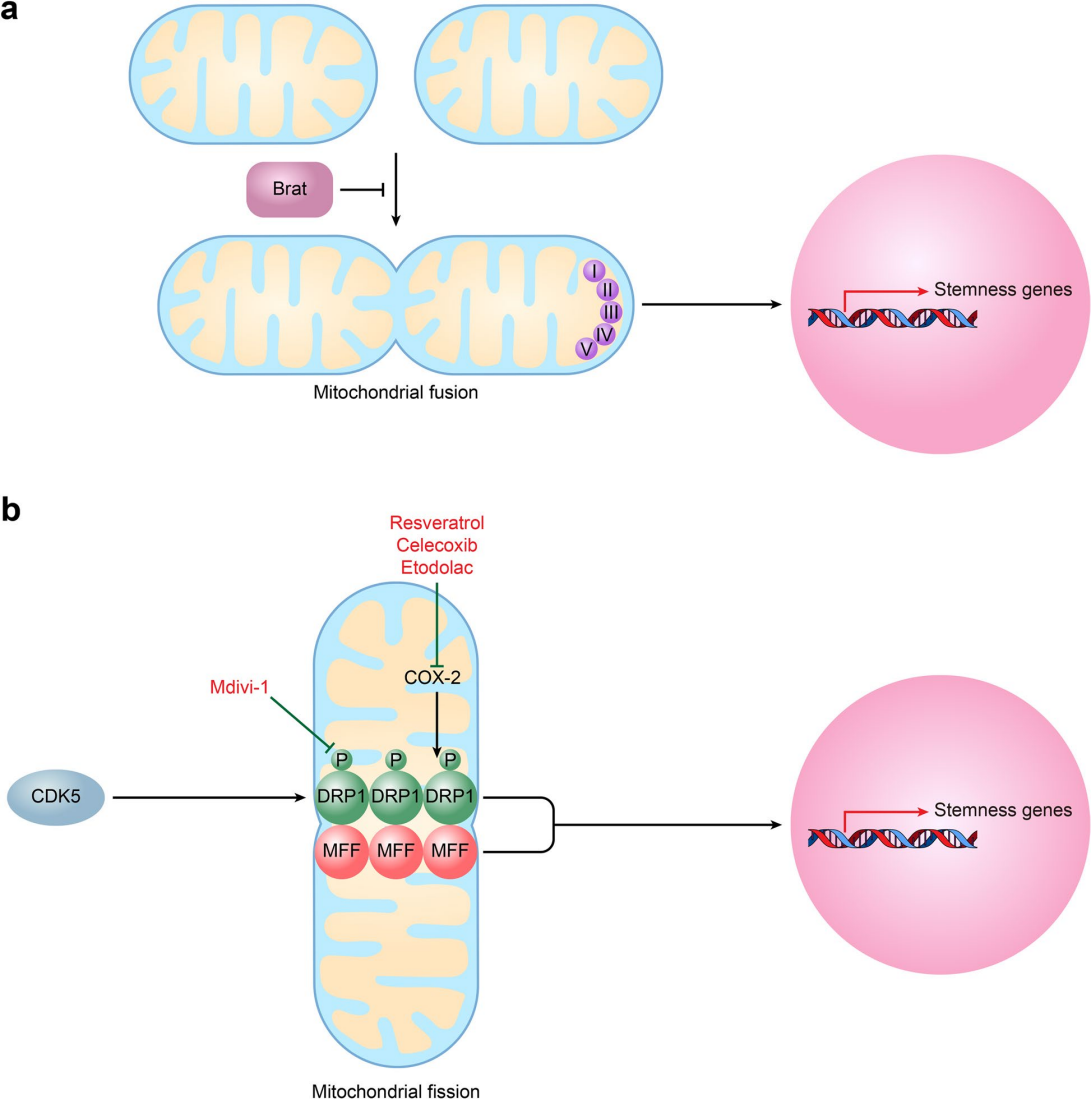
Mitochondrial dynamics and cancer stem cell stemness. **A** Mitochondrial fusion induced by brat knockdown increased OXPHOS and NAD^+^ levels and then drove the immortalization and tumorigenicity of tumor-initiating cells (TICs). **B** CDK5 phosphorylates DRP1 and increases mitochondrial cleavage, which then induces the expression of stemness genes. Similarly, MFF promotes mitochondrial fission and then increases OCT4 expression levels. Mdivi-1 reduces the expression of stemness genes by inhibiting DRP1; selective COX-2 inhibitors resveratrol, celecoxib, and etodolac reduce the expression level of stemness genes by inhibiting DRP1 indirectly. *OXPHOS* Oxidative phosphorylation, *TICs* Tumor-initiating cells, *CDK5* Cycle-dependent kinase 5, *MFF* Mitochondrial fission factor

Both fusion and fission can enhance CSC stemness and maintain self-renewal, depending on the tissue type of the tumor. Bonnay *et al* found that mitochondrial fusion helps to sustain CSC fates or phenotypes in neuroblastoma [89]. In detail, mitochondrial fusion induced by brat knockdown increased oxidative phosphorylation (OXPHOS) and NAD^+^ levels and then drove the immortalization and tumorigenicity of TICs [89] (Fig. 3A). However, some other studies have indicated that mitochondrial fission promotes the potential and self-renewal of CSCs [90]. In liver cancer, the complex formed by T-box transcription factor 19 (TBX19) and PRMT1 induces MFF expression by increasing histone H4R3me2a/H3K9ac levels; then, MFF promotes mitochondrial fission, increases the expression level of OCT4, enhances the formation of tumorspheres and enriches the side populations (SPs) [91] (Fig. 3B). In glioblastomas, cycle-dependent kinase 5 (CDK5) phosphorylates DRP1 at Ser616 and increases mitochondrial cleavage, which then induces the expression of stemness genes (*OLIG2*, *OCT4*, *NANOG*, *NESTIN*, *POU3F2*, *CD133*, *SSEA1*) [92] (Fig. 3B). Contrary to popular views, recent studies have shown evidence that mitochondrial fission inhibits the stemness of CSCs. For example, the activation of mitochondrial fission by overexpressing MFF impairs BCSC propagation through methods such as reducing the capacity for tumorsphere formation and the enzyme activity of the stem cell marker ALDH [93].

### Mitochondrial homeostasis

Mitochondrial biogenesis and mitophagy are two balanced processes in cells that control the quantity and quality of mitochondria [94]. Mitochondrial biogenesis is the process of replenishing new healthy mitochondria, while mitophagy is the activity by which cells spontaneously phagocytose or selectively degrade dysfunctional or redundant aging mitochondria under conditions of stress such as hypoxia or nutritional deficiency [95–98]. Mitochondrial biogenesis and mitophagy are both key events in regulating the stemness of CSCs.

**Fig. 4:**
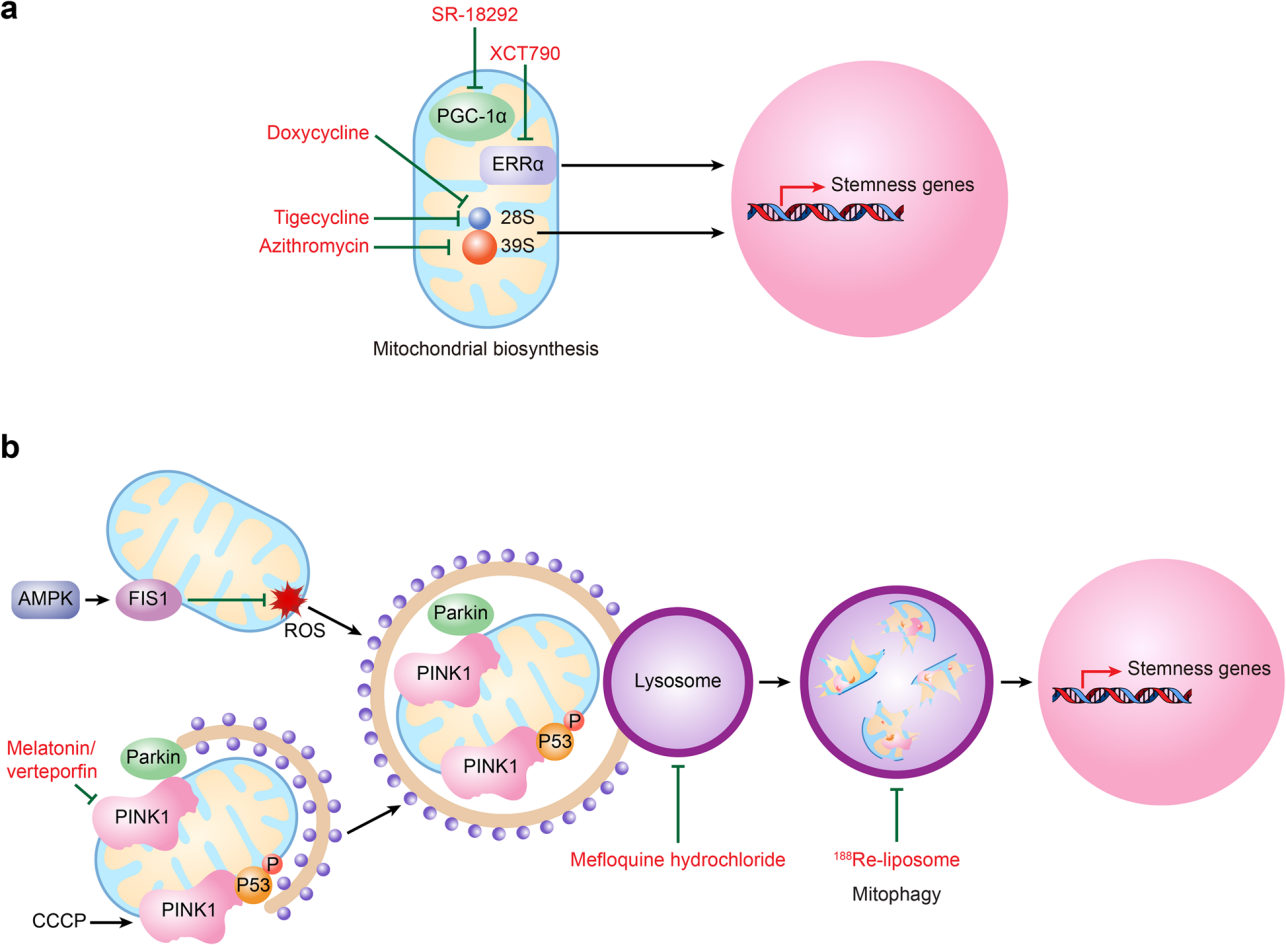
Mitochondrial homeostasis and cancer stem cell stemness. **A** PGC-1α or its cofactor ERRα increases mitochondrial biogenesis, which further promotes the maintenance of the CSC phenotype. Azithromycin, doxycycline, and tigecycline block mitochondrial biogenesis by targeting mitochondrial ribosomes 39S and 28S and then inhibit CSC self-renewal capability. In addition, the selective PGC-1α inhibitor SR-18292 or ERRα inverse agonist XCT790 downregulates the expression of stemness genes and reduces the ratio of CSCs. **B** AMPK-FIS1 signaling promotes mitophagy, thereby enhancing CSC self-renewal by inhibiting ROS production. CCCP increases CSC ratios by recruiting PINK1 and enhancing mitophagy-mediated removal of phosphorylated p53 and then increases NANOG expression. In contrast, the combination of melatonin and verteporfin reduces CSC stemness by inhibiting the expression of PINK1/Parkin, while mefloquine hydrochloride reduces the ratio of colon CSCs by inhibiting mitophagy and lysosomal activity. In addition, ^188^Re-liposomes reduced the protein levels of mitophagy markers, which further decreased the function of CSCs. *CSC* Cancer stem cell, *AMPK* Adenosine 5'-monophosphate-activated protein, *FIS1* Fission factor 1, *CCCP* Carbonyl cyanide chlorophenylhydrazone, *PINK1* PTEN-induced kinase 1

Promoting mitochondrial biogenesis by PGC-1α [99] or its cofactor estrogen-related receptor α (ERRα) [100] increases tumorsphere formation in pancreatic cancer and breast cancer, respectively (Fig. 4A). In contrast, PGC-1α knockdown in glioblastoma cells leads to the attenuation of the neoplastic phenotype and loss of stem-like features, which was reflected by reduced expression of the stemness gene *SOX2* but prolonged survival of nude mice [101].

Regarding mitophagy, an increase in mitophagy by the depression of oxidative stress or an attenuation of mitophagy by parkin interference can lead to the generation or loss of CD44^high^CD24^-/low^ esophageal CSCs [102]. For example, adenosine 5'-monophosphate-activated protein (AMPK)-FIS1 signaling-mediated mitophagy leads to the elimination of damaged mitochondria, thereby inhibiting intracellular ROS production and promoting the self-renewal and survival of LSCs [103] (Fig. 4B). At the molecular level, mitophagy promotes the formation of CSCs by altering the subcellular location of phosphorylated p53 between mitochondria and the nucleus. In detail, when mitophagy is enhanced by carbonyl cyanide chlorophenylhydrazone (CCCP), PTEN-induced kinase 1 (PINK1) recruits and phosphorylates p53 at serine-392 and entraps p53 on mitochondria; subsequently, p53 is removed by mitophagy, and thus, the expression of NANOG and the ratio of the CD133^+^ liver CSC population are increased (Fig. 4B). Conversely, when mitophagy is inhibited by a mitochondrial fission inhibitor (Mdivi-1), PINK1-phosphorylated p53 is rapidly translocated to the nucleus, which results in the suppressed expression of NANOG and a reduction in the CD133^+^ CSC ratio [104].

### Mitochondrial ROS

**Fig. 5:**
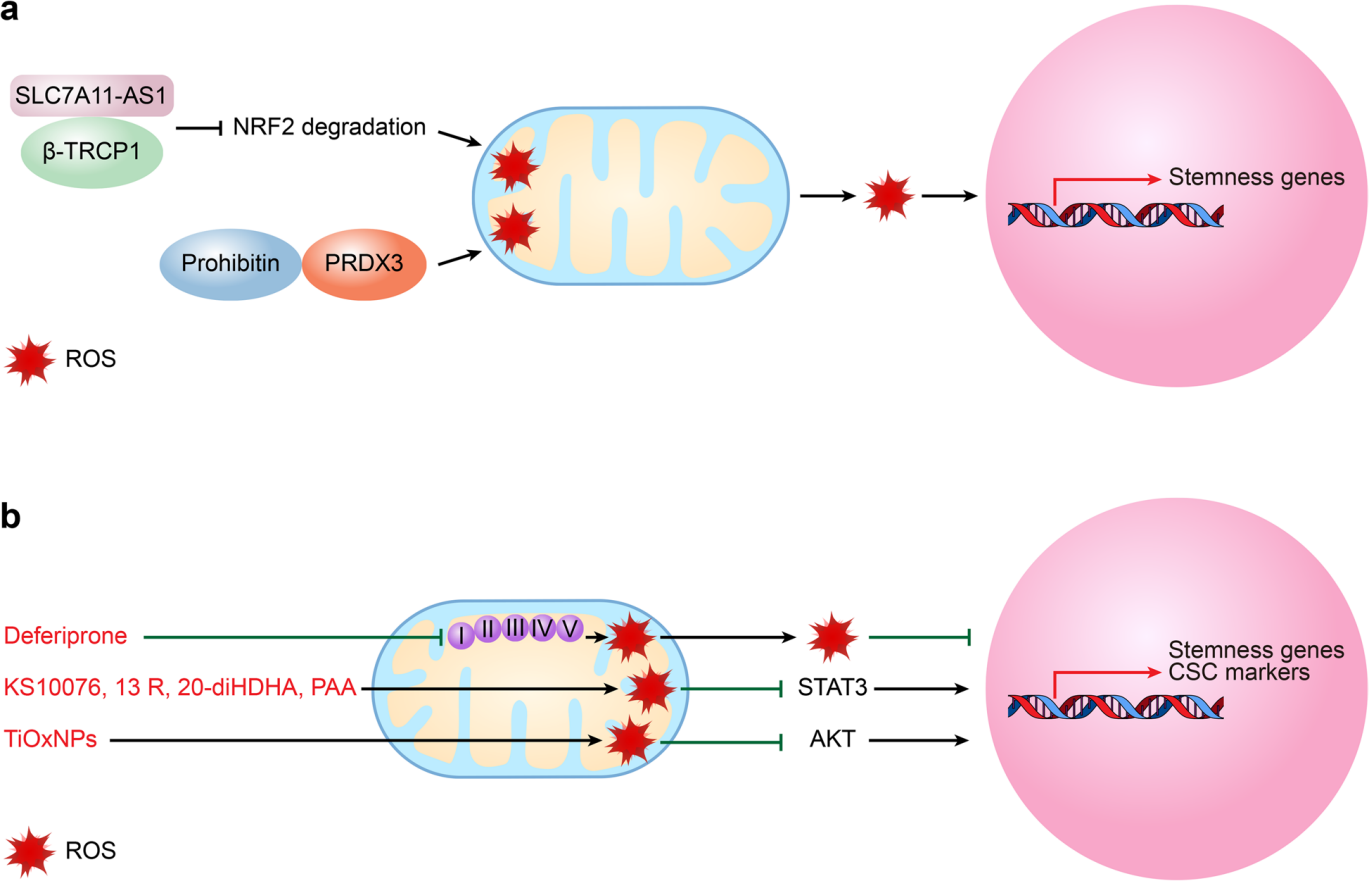
Mitochondrial ROS signaling and cancer stem cell stemness. **A** SLC7A11-AS1/β-TRCP1 or prohibitin promote CSC stemness potential by blocking NRF2 degradation and thus scavenging ROS or by interacting with PRDX3 and maintaining low mitochondrial ROS levels. **B** By blocking the role of iron in electron transport, deferiprone induces mitochondrial ROS and disrupts CSC stemness. KS10076, 13 R, 20-diHDHA, and PAA induce the production of ROS, degrade STAT3 or decrease the expression levels of CSC self-renewal genes, while TiOxNPs reduce CSC marker expression levels by inducing ROS levels and inactivating AKT signaling. *CSC* Cancer stem cell, *13 R* 20-diHDHA, *13R* 20-dihydroxydocosahexaenoic acid, *PAA* Phenylacetaldehyde, *TiOxNPs* Titanium peroxide nanoparticles

ROS, the main byproducts of oxidative metabolism, include superoxide radical anions, hydroxyl radicals, hydrogen peroxide, and lipid hydrogen peroxide [105, 106]. The roles of ROS in regulating CSC self-renewal and survival occur in a context- and tissue-dependent manner. Generally, ROS are reported to maintain CSC properties and to induce CSC proliferation and tumorigenicity. In colorectal cancer (CRC), RAC1 activation upon Apc loss triggers the production of high levels of ROS in the intestines of *vil-Cre-ER*^*T2*^* Apc*^*fl/fl*^* Rac1*^*fl/fl*^ mice; then, ROS further increase the expression of stemness genes (*LGR5*, *OLFM4*, *RGMB*), confer LGR5 CSC phenotypes, and thus initiate CRC [107]. Conversely, as ROS levels decrease following a sublethal dose of H_2_O_2_, ESA^+^CD44^+^CD24^*-*^ BCSCs lose the ability to form tumorspheres and colonies [108]. However, many studies have found that increasing intracellular ROS levels can induce CSC death and that a low level of ROS is required for the maintenance of cancer stemness [109]. In pancreatic cancer, lncRNA SLC7A11-AS1 promoted stemness potential by scavenging ROS, which functioned by interacting with β-TRCP1 and then blocking nuclear factor erythroid-2-related factor 2 (NRF2) degradation [110] (Fig. 5A). In GSCs, high levels of prohibitin promote GSC self-renewal by stabilizing PRDX3 and maintaining low levels of mitochondrial ROS [111] (Fig. 5A).

## Blocking mito-nuclear communication as an emerging strategy for anti-CSC therapy

Despite significant improvements in anticancer drug development, CSC-derived chemoresistance and recurrence are still major challenges for cancer treatments. As CSCs and non-CSCs have different metabolic characteristics and mitochondrial metabolism governs CSC fates, blocking mito-nuclear communication would be an effective and innovative strategy. In the following section, we summarize the recent progress in gene interventions and therapeutic agents targeting mito-nuclear communication for CSC eradication (Tables 1 and 2).

**Table 1:** Genetic inhibition for blocking mito-nuclear communication in CSCs

Patterns of mito-nuclear communication	Names of genes	Mechanisms of action	CSC types and tissue origins
Mitochondrial fission	DRP1	Reduces the capability of tumorsphere and tumor formation, especially the self-renewal of CSCs	CD133^+^CD15^+^ BTICs [92]
	MFF or BRD4	Reduces the capability of tumorsphere and tumor formation, especially the self-renewal of CSCs	Prostate CSCs [112]
	FIS1	Reduces the expression levels of stemness genes and inhibits tumorsphere and tumor formation, especially the self-renewal of CSCs	sphere enriched Lung CSCs [113]
Mitophagy	PINK1 and TBC1D15	Decreases the levels of self-renewal markers	LSCs [103]
	ATG14	Decreases the expression levels of self-renewal and stemness markers and the number of tumorspheres	CSCs in oral squamous cell carcinoma [114]
	BNIP3L	Decreases the expression levels of stemness markers and the percentage of SP^+^ subpopulations	LCSCs [115]

**Table 2:** Therapeutic agents for blocking mito-nuclear communication in CSCs

Patterns of mito-nuclear communication	Key mitochondrial behaviors and targets	Therapeutic agents	Mechanisms of action	CSC types and tissues origin
Mitochondrial energy metabolism	Oxidative phosphorylation, mitochondrial complex I	Metformin, mitochondrial complex I inhibitor	Downregulates the expression of CSC-related genes, decreases the ratios of CD44^high^ALDH^high^ cells as well as the sizes and numbers of tumorspheres, reduces the volume of tumors	sphere enriched CSCs in cholangiocarcinoma [116]; HNSCC CSCs [117]
	Methionine cycle, SAM levels	FIDAS-5, MAT2A inhibitor	Inhibits the expression of methylation marks of H3K4me3 etc*.*, reduces the mass and volume of xenograft tumors, diminishes the size of NOD.Cg-Prkdc^scid^ Il2rgtm1^Wjl/SzJ^ mouse lung lesions, decreases the ratio of CD166^+^ CSCs	sphere enriched and CD166^+^ Lung CSCs [118]
		Cycloleucine, MAT2A inhibitor	Suppresses the demethylation of H3K4me3 and inhibits the protein expression of the stemness transcription factor SOX9; the combination of cycloleucine and methionine depletion more effectively reduces mammospheres in vitro and the burden of primary and lung metastases in vivo	sphere enriched BCSCs [119]
Mitochondrial dynamics	Mitochondrial fission	Mdivi-1, DRP1 inhibitor	Reduces the percentages of SP^+^ and CD44^+^ CSC subpopulations, inhibits the expression of stemness genes, suppresses the formation capacity of tumorspheres in vitro and tumors in vivo, and decreases the ability of self-renewal	LCSCs [91]; NPC CSCs [120]; Ovarian CSCs and Colorectal CSCs [121]; Pancreatic CSCs [86]; CD133^+^CD15^+^BTICs [92]; EpCAM^+^CD133^+^ LCSCs [91]
		Resveratrol, etodolac, celecoxib; selective COX-2 inhibitor	Reduces the expression levels of the stemness genes, the ratio of SP^+^ subpopulations, and the capacity for tumorsphere formation	NPC CSCs [120]; Bladder CSCs [122]; Glioblastoma CSCs [123];
	MFF	OTX015, BRD4 inhibitor	Suppresses tumorigenicity and self-renewal ability	Prostate CSCs [112]
		Furamidine, PRMT1 inhibitor	Blocks TBX19-induced mitochondrial fission, and decreases the capacity of tumorsphere formation and tumorigenesis	LCSCs [91]
Mitochondrial homeostasis	Mitochondrial mitophagy	Combination of melatonin and verteporfin, PINK1/parkin signa lling pathway inhibitor	Reduces the capacity of tumorsphere formation and the numbers of CD44^+^CD24^−^ and CD133^+^ CSCs	HNSCC CSCs [124]
		Mefloquine hydrochloride, lysosomes RAB5/7 inhibitor	Decreases the ratio of CD44v9^+^/CD133^+^ colon CSCs	CD44v9^+^/CD133^+^ Colon CSCs [125]
		^188^Re-liposome, nanomedicine, lysosomal proteins inhibitor	Reduces the protein levels of (Lamp-1 and cathepsin-B) and autophagy/mitophagy (LC3B, Atg16 L and Becline-1) markers, decreases tumor growth in xenograft mouse models, lowers CA-125 levels, and prolongs ovarian cancer patients’ survival in a clinical phase I trial	Ovarian CSCs [126, 127]
	Mitochondrial biosynthesis	Azithromycin, doxycycline; Tigecycline, mitochondrial ribosome inhibitor	Targets the 39 s and 28 s mitochondrial ribosomes and inhibits tumorsphere formation, tumorigenicity and self-renewal ability	CSCs in breast, ovarian, lung, prostate, pancreatic cancer, melanoma, DCIS and GBM [128]; LSCs [129]
		SR-18292, selective PGC-1α inhibitor	Reduces tumorsphere formation, shrinks tumor size, and downregulates the expression of genes involved in stemness maintenance and self-renewal	sphere enriched CSCs in cholangiocarcinoma [116]
		XCT790, ERRα-PGC1 signaling pathway inhibitor	Inhibits the formation of mammospheres and decreases the proportion, survival and propagation of CD44^+^CD24^−^ BCSCs	BCSCs [100]
Mitochondrial ROS	Electron transport	Deferiprone, iron chelator	Decreases the proportions of tumorspheres and ALDH^+^ CSCs	BCSCs [130]
	ROS-STAT3 signaling pathway	KS10076, metal chelator	Reduces the capacity for tumor formation, decreases the expression levels of CSC self-renewal genes, and decreases the ratios of subpopulations of ALDH^+^ or CD44^high^CD24^low^ CSCs	Colon CSCs [131]
		13 R, 20-diHDHA, dihydroxy-DHA derivative	Reduces the capacity for tumorsphere formation, decreases the expression levels of CSC self-renewal genes, and decreases the ratios of subpopulations of ALDH^+^ or CD44^high^CD24^low^ BCSCs	sphere enriched BCSCs [132]
		PAA, flower flavor	Reduces the capacity for tumorsphere formation and tumor formation, decreases the expression levels of CSC self-renewal genes, and decreases the ratios of subpopulations of ALDH^+^ or CD44^+^CD24^−^ CSCs	sphere enriched BCSCs [133]
	ROS-AKT signaling pathway	TiOxNPs, titanium peroxide nanoparticles	Sensitizes radioresistant CSCs to ionizing radiation, decreases tumorsphere number and CSC marker expression levels, reduces the pancreatic CSC self-renewal ability, decreases tumor growth rate and necrosis area, and improves of mouse survival rate	sphere enriched Pancreatic CSCs [134]

### Targeting mitochondrial energy metabolism

The well-known means of disrupting the maintenance of CSC features by interfering with mitochondrial energy metabolism is the inhibition of mitochondrial complex I of oxidative phosphorylation. One such example is the effective drug metformin, which downregulates the expression of CSC-related genes, decreases the ratios of CD44^high^ALDH^high^ cells as well as the sizes and numbers of tumorspheres, and thus reduces the volume of tumors in cholangiocarcinoma and head and neck squamous cell cancer (HNSCC) [116, 117] (Fig. 2B).

In addition, mitochondrial-derived metabolites are popular choices for targeting CSCs by interfering with mitochondrial energy metabolism. For example, SAM is the universal donor for DNA and histone methylation and has been linked to CSC self-renewal; therefore, decreasing SAM levels by blocking the methionine cycle key enzyme adenosyltransferase 2A (MAT2A) is considered an ideal strategy for eradicating CSCs. In lung cancer, it was reported that the MAT2A inhibitor FIDAS-5 strongly decreases the intracellular level of SAM and significantly inhibits the expression of methylation marks such as H3K4me3, H3K9me3, H3K27me3, H3K36me2, H3K36me3 and H3K79me3 in CSCs, thereby greatly lowering the tumorigenic potential of CSCs, which includes reducing the mass and volume of xenograft tumors and diminishing the size of NOD. Cg-Prkdc^scid^ Il2rgtm1^Wjl/SzJ^ mouse lung lesions and decreased the ratio of CD166^+^ CSCs [118] (Fig. 2B). Another MAT2A inhibitor, cycloleucine, has been found to enhance the suppressive effect of methionine depletion on BCSCs by inhibiting the protein expression of the stemness transcription factor SOX9 by suppressing the demethylation of H3K4me3 (Fig. 2B). In addition, the combination of MAT2A inhibition and methionine depletion could more effectively reduce mammospheres *in vitro* and the burden of primary and lung metastases *in vivo* [119]*.*

### Targeting mitochondrial dynamics

Some studies have found that inhibiting mitochondrial fission-related proteins may be another potential therapeutic strategy for targeting the stemness potential of CSCs. For example, knockdown of mitochondrial fission-related genes, such as *DRP1*, *MFF*, *BRD4* or *FIS1*, reduces the expression levels of stemness genes and the capability of forming tumorspheres and tumors, especially the self-renewal of CSCs in brain, prostate and lung cancers [92, 112, 113] (Table 1).

For pharmacological intervention, Mdivi-1 is the most common and generally recognized DRP1-selective inhibitor that has been reported to reduce the percentage of SP^+^ or CD44^+^ CSC subpopulations, the expression of stemness genes, and the formation capacity of tumorspheres *in vitro* and in tumors *in vivo* in the context of nasopharyngeal carcinoma (NPC), liver cancer, ovarian cancer, *etc.* [86, 91, 120, 121] (Fig. 3B). Moreover, Mdivi-1 directly suppresses the function of CSCs that are isolated by stem cell markers. In detail, Mdivi-1 inhibits the self-renewal and tumor initiation capacities of CD133^+^CD15^+^ brain tumor-initiating cells (BTICs) [92] and decreases the levels of stemness genes in EpCAM^+^CD133^+^ liver cancer stem cells (LCSCs) [91]. As COX-2 maintains the CSC phenotype by activating DRP1, selective COX-2 inhibitors, such as resveratrol, celecoxib, and etodolac, are reported to reduce the expression levels of stemness genes, the ratio of SP^+^ subpopulations, and the tumorsphere formation capacity in NPC, bladder cancer, and glioblastoma [120, 122, 123] (Fig. 3B). In addition, BRD4 or PRMT1 can act as a transcription regulator or an epigenetic activator for MFF, respectively; therefore, targeting BRD4 or PRMT1 can be an attractive therapeutic option for eradicating CSCs by blocking mitochondrial fission. Therefore, the BRD4 inhibitor OTX015 suppresses tumorigenicity and self-renewal ability in prostate CSCs, and the PRMT1 inhibitor furamidine blocks TBX19-induced mitochondrial fission and decreases the capacity for tumorsphere formation and tumorigenesis in LCSCs [91].

### Targeting mitophagy or mitochondrial biosynthesis.

Because mitophagy actively promotes the production of CSCs, targeting mitophagy-related genes could counteract CSCs. In AML, oral squamous cell cancer, and liver cancer, the knockdown of mitophagy-related regulator genes, such as *PINK1*, *TBC1D15*, *ATG14*, and *BNIP3*, decreases the levels of self-renewal and stemness, the number of tumorspheres, and the percentage of SP^+^ subpopulations [103, 114, 115] (Table 1).

Unfortunately, there are few reports on targeting CSCs by mitophagy-specific inhibitors but indirect mitophagy inhibition. For example, the combination of the circadian rhythm-regulating molecule melatonin and the YAP/TAZ inhibitor verteporfin was shown to decrease the expression of PINK1/parkin and then to reduce the capacity for tumorsphere formation and the numbers of CD44^+^CD24^-^ and CD133^+^ CSCs in HNSCC [124] (Fig. 4B). However, some reports have revealed that targeting mitophagy-associated lysosomes could interfere with CSC function. As RAB5/7 (the regulators of early and late lysosome biogenesis) and LAMP1/2 (lysosomal/late endosomal marker and lysosomal receptor, respectively) were able to facilitate PINK1/parkin-dependent mitophagy, targeting RAB5/7 or LAMP1/2 could be a potential strategy for eradicating CSCs (Fig. 4B). Mefloquine hydrochloride, a novel RAB5/7 inhibitor, was reported to disrupt CD44v9^+^CD133^+^ colon CSCs by inhibiting lysosomal activity and mitophagy and thus could be a promising colorectal CSC-targeting drug [125] (Fig. 4B). In addition, the nanomedicine ^188^Re-liposome was shown to effectively suppress the expression of stemness markers and reduce the protein levels of lysosome (Lamp-1 and cathepsin-B) and autophagy/mitophagy (LC3B, Atg16L and Beclin-1) markers, which then resulted in decreased tumor growth in xenograft mouse models, lowered CA-125 levels and prolonged ovarian cancer patient survival in a clinical phase I trial [126, 127] (Fig. 4B).

In contrast to inhibitors of mitophagy, many antibiotics that inhibit mitochondrial biogenesis have become a prospective means of depleting CSCs. Azithromycin, doxycycline, and tigecycline, which target the 39S and 28S mitochondrial ribosomes, were shown to inhibit tumorsphere formation, tumorigenicity and self-renewal ability in breast cancer, AML, *etc.* [128, 129] (Fig. 4A). More specifically, drugs targeting PGC-1α and related genes are another attractive therapeutic choice for CSC elimination. SR-18292, a selective PGC-1α inhibitor, reduces tumorsphere formation, shrinks tumor size, and downregulates the expression of genes involved in stemness maintenance and self-renewal in cholangiocarcinoma [116] (Fig. 4A). XCT790, an ERRα inverse agonist, inhibits the formation of mammospheres in a concentration-dependent manner and decreases the percentage, survival and propagation of CD44^+^CD24^-^ BCSCs [100] (Fig. 4A).

### Induction of mitochondrial ROS production

CSCs maintain mitochondrial ROS at a low level; thus, inducing mitochondrial ROS is considered a novel option for anti-CSC-based therapy. Due to the critical roles of iron in electron transport and ROS generation, an iron chelator is considered a candidate for eradicating CSCs. FDA-approved deferiprone is such an example that dose-dependently decreases the tumorsphere numbers of CSCs and ALDH^+^ BCSCs by inducing mitochondrial ROS and is now being recommended for phase II clinical trials [130] (Fig. 5B). By inducing ROS-mediated STAT3 degradation or downregulation, the metal chelator KS10076, a novel dihydroxy-DHA derivative 13R, 20-dihydroxydocosahexaenoic acid (13 R, 20-diHDHA), and flower flavor phenylacetaldehyde (PAA) were reported to be potential agents for targeting CSCs by reducing the size of the tumorsphere and tumor formation, decreasing the expression of CSC self-renewal genes, and decreasing the ratios of subpopulations of ALDH^+^ or CD44^high^CD24^low^ (CD44^+^CD24^−^) CSCs in colon and breast cancers [131–133] (Fig. 5B). In addition, by producing intolerable levels of ROS and inactivating AKT signaling, titanium peroxide nanoparticles (TiOxNPs) sensitize radioresistant CSCs to ionizing radiation through the decline of tumorsphere number and CSC marker expression and the reduction of pancreatic CSC self-renewal ability, which then leads to decreases in tumor growth rate and necrosis area while improving mouse survival rate [134] (Fig. 5B).

## Conclusions and perspectives

Mitochondria have emerged as a regulatory hub of energy and signaling that can alter the fate of CSCs through the mito-nuclear communication process described above. Therefore, targeting mito-nuclear communication would eradicate CSCs and provide therapeutic benefits for cancer patients. To date, although a few studies that focus on blocking mito-nuclear communication have made great breakthroughs in inhibiting CSC potential, there are some challenges that still need to be overcome in the future.

First, the functional activities of the mitochondria are essential to both normal cells and CSCs. Thus, normal cells may be impaired by therapeutic agents that target mitochondria, which results in some side effects. However, the sensitivity of normal cells and CSCs to drugs may be different. More importantly, real-time monitoring of drug concentrations and metabolite levels would help to improve the specificity of targeting CSC agents and to reduce their toxicity to normal cells.

Second, during the process of tumorigenesis, the metabolic requirements of cancer cells change continuously. As these changes are caused by the input and changes in microenvironment signals, we need to target microenvironment signaling and CSCs together. Normal cells and CSCs may have different responses to changes in microenvironment signaling; thus, it is possible to eradicate CSCs while avoiding damaging normal cells.

## Data Availability

Not applicable.

## Abbreviations

CSCs: Cancer stem cells
ROS: Reactive oxygen species
TSCs: Tumor stem-like cells
CICs: Cancer initiating cells
TICs: Tumor-initiating cells
MeCSC: Metastatic cancer stem cell
CRCSC: Chemo-resistant cancer stem cell
GBM: Glioblastoma
GC: Gemcitabine and cisplatin
PGE2: Prostaglandin E2
BCSCs: Breast cancer stem cells
EMT: Epithelial-to-mesenchymal transition
MET: Mesenchymal-to-epithelial transition
ALDH^+^: Aldehyde dehydrogenase positive
HCC: Hepatocellular carcinoma
TFs: Transcription factors
GSCs: Glioblasoma stem cells
ZHX2: Zinc-finger and homeoboxe 2
mtDNA: Mitochondrial DNA
acetyl-CoA: Acetyl-coenzyme A
βHB: β-Hydroxybutyric acid
SAM: S-adenosylmethionine
*α*-KG: *α*-Ketoglutarate
AML: Acute myeloid leukemia
LSC: Leukemia stem cell
TKI: Tyrosine kinase inhibitor
DRP1: Dynamin-related protein 1
MFF: Mitochondrial fission factor
FIS1: Fission factor 1
MFN1: Mitofusin 1
MFN2: Mitofusin 2
OPA1: Optic atrophy 1
OXPHOS: Oxidative phosphorylation
TBX19: T-box transcription factor 19
SP: Side population
CDK5: Cyclin-dependent kinase 5
ERRα: Estrogen-Related Receptor α
CCCP: Carbonyl cyanide m-chlorophenylhydrazone
PINK1: PTEN-induced kinase 1
Mdivi-1: Mitochondrial fission inhibitor
AMPK: Adenosine 5'-monophos-phate-activated protein kinase
CRC: Colorectal cancer
NRF2: Nuclear factor erythroid-2-related factor 2
NPC: Nasopharyngeal carcinoma
BTICs: Brain tumor-initiating cells
LCSCs: Liver cancer stem cells
13 R: 20-DiHDHA: 13R: 20-dihydroxydocosahexaenoic acid
MAT2A: Methionine adenosyltransferase 2A
STAT3: Signal transducer and activator of transcription 3
HNSCC: Head and neck squamous cell carcinoma
